# Flexible Ureteroscopy Can Be More Efficacious in the Treatment of Proximal Ureteral Stones in Select Patients

**DOI:** 10.1155/2015/416031

**Published:** 2015-11-04

**Authors:** Erdal Alkan, Ali Sarıbacak, Ahmet Oguz Ozkanli, Mehmet Murad Basar, Oguz Acar, Mevlana Derya Balbay

**Affiliations:** ^1^Department of Urology, Memorial Şişli Hospital, Şişli, Istanbul, Turkey; ^2^Department of Urology, Konak Hospital, Izmit, Turkey; ^3^Department of Anesthesiology, Memorial Şişli Hospital, Şişli, Istanbul, Turkey

## Abstract

*Purpose*. We aimed to compare and evaluate the outcomes and complications of two endoscopic treatment procedures, semirigid ureteroscopy (SR-URS) and flexible ureteroscopy (F-URS), in the treatment of proximal ureteral stones (PUS). *Methods*. SR-URS (group 1) was done on 68 patients whereas 64 patients underwent F-URS (group 2) for the treatment of PUS. Success rate was defined as the absence of stone fragments or presence of asymptomatic insignificant residual fragments < 2 mm. Outcomes and complications were recorded. *Results*. The differences were statistically not significant in age, gender, body mass index (BMI), and stone characteristics between groups. Mean ureteral stone size was 9.1 ± 0.4 mm and 8.9 ± 0.5 mm for groups 1 and 2. Mean operative time was 34.1 ± 1.5 min and 49.4 ± 2.3 min for groups 1 and 2 (*p* = 0.001). SFRs were 76.5% and 87.5% for groups 1 and 2 (*p* = 0.078). Two major complications (ureteral avulsion and ureteral rupture) occurred in group 1. *Conclusion*. F-URS is safer and less invasive than SR-URS in patients with PUS. There is no statistically significant difference in the efficacy of either technique. Nonetheless we recommend F-URS in the management of PUS as a first-line treatment option in select cases of proximal ureteral calculi.

## 1. Introduction

There are various options in the management of proximal ureteral stones (PUS), which includes medical expulsive therapy (MET), extracorporeal shock wave lithotripsy (ESWL), ureteroscopy (URS; retrograde), percutaneous nephrolithotomy (PCNL), laparoscopy (LAP), and open surgery [1]. Nowadays, ESWL and URS are the most commonly performed treatment options in the management of PUS. Although 2014 update of the European Association of Urology (EAU) urolithiasis guidelines showed that both URS and ESWL should be considered as a first-line therapy for PUS, the optimal treatment of these stones still remains debatable [1]. With the development of endoscopic equipment especially holmium laser and small caliber ureteroscopes, and accumulation of experience, ureteroscopic lithotripsy especially flexible ureteroscopy has been widely used [1, 2].

There are many studies comparing the above-mentioned treatment modalities in the management of PUS [2–6]. However, to our knowledge there is only one study comparing semirigid URS (SR-URS) and flexible URS (F-URS) [7]. We retrospectively compared and evaluated the outcomes and complications of rigid and flexible URS for the treatment of PUS.

## 2. Materials and Methods

### 2.1. Patients

Medical reports were retrospectively reviewed for patients with PUS who underwent ureteroscopy (SR-URS or F-URS) between February 2007 and January 2015. All patients were evaluated by CT scan with stone protocol prior to the operation. Each patient was evaluated for body mass index (BMI), stone location, stone number, stone size (assessed by measuring its largest dimension in CT imaging), stone burden (cumulative stone length of the stones), operative time, hospital stay, stone free rates (SFR), and perioperative complications. Inclusion criteria included adult patients (≥20 years) and patient having PUS with or without small renal stones (≤10 mm). Nevertheless all stones regardless of their size within the proximal ureter were included. Exclusion criteria included acute urinary tract infection, operation history of ipsilateral ureter or kidney, congenital ureteropelvic junction obstruction, coexisting ureteral disorders (including tumor or stricture), and patients with simultaneous middle or lower ureteral stones. Patients who had internal stent preoperatively were also excluded. Additionally, when the renal stones were larger than 10 mm, these patients were also excluded regardless of proximal ureteric stone size. Ureteroscopy was indicated and preferred by these patients due to failed ESWL, obesity, and patient preference. Patients with PUS ≤ 5 mm were treated with medical expulsive therapy for 3 weeks in each group. At the end of this period, an ureteroscopic lithotripsy was planned since spontaneous passage of the stones did not occur.

The patients were divided into two groups: patients who underwent SR-URS and F-URS were included in group 1 (*n* = 68) and group 2 (*n* = 64), respectively. F-URS had been mainly preferred in the following conditions due to institutional policy: (1) PUS together with concomitant renal stone, (2) patients with grade 3 or 4 hydroureteronephrosis, and (3) less than 5 cm distance from ureteropelvic junction to the ureteral stone. Patients with PUS only were treated with semirigid ureteroscopy when the ureteric stone was located further than 5 cm from the ureteropelvic junction. This area can be easily reached with semirigid ureteroscopy which limits the use of more expensive flexible ureteroscopy and extends its life span. Stone clearance was assessed intraoperatively and checked with CT or urinary US at postoperative 3 months. Success rate was defined as the absence of stone fragments or presence of asymptomatic insignificant residual fragments <2 mm. Perioperative complications were recorded according to the Clavien-Dindo classification system [8]. All procedures were performed by three experienced surgeons with similar indication and the same surgical techniques.

### 2.2. Techniques 

#### 2.2.1. Rigid Ureteroscopic Lithotripsy

Using a urological guide wire, a 7.5 F semirigid ureteroscope (Karl Storz, Germany) was inserted into the ureter. A stone cone (Stone Cone Nitinol Retrieval Coil, 3.0 F × 115 cm × 7 mm coil; Boston Scientific, Natick, MA, USA) was advanced beyond the stone and fragmented into small pieces by holmium laser (Sphinx, Lisa Laser, 30 watts, Katlenburg-Lindau, Germany) using 365 *μ*m (PercuFib, Lisa Laser, Katlenburg-Lindau, Germany) laser fibers. Larger stone fragments were removed by endoscopic forceps. At the end of the procedure, a 4.8 F 26 cm internal stent was inserted based on surgeon's decision. When the stone was pushed back to the kidney, the procedure was completed using flexible ureteroscope, and rigid ureteroscopy was accepted as unsuccessful. These patients were not included in group 2. In case semirigid ureteroscope could not be advanced up to the proximal ureter due to ureteral stricture, an internal stent was inserted into the ureter and the intervention was delayed at least 15 days. This procedure was also accepted as unsuccessful.

#### 2.2.2. Flexible Ureteroscopic Lithotripsy

An access sheath (Flexor ureteral access sheath 12/14 F 35 cm; FUS, Cook Medical, Bloomington, IN, USA) was introduced into the proximal ureter over a 0.038-inch safety hydrophilic guide wire (Sensor, Microvasive, Boston Scientific Corp, Natick, MA, USA). URF P-5 flexible ureteroscope (Olympus, Tokyo, Japan) and Cobra Flexible Dual-Channel Ureteroscope (Richard Wolf, Knittlingen, Germany) were used in all cases according to their availability. The stone was fragmented with holmium laser (Sphinx, Lisa Laser, 30 watts, Katlenburg-Lindau, Germany) in combination with 200 *μ*m or 272 *μ*m (LithoFib and FlexiFib, Lisa Laser, Katlenburg-Lindau, Germany) laser fibers in the proximal ureter. In case the stone was pushed back to the collecting system, the stone was fragmented in the kidney. When required, a nitinol basket (Ngage nitinol stone extractor 2,2 F 115 cm basket; Cook Medical Bloomington, IN, USA) was used for the removal of stone fragments. Endoscopically, intraoperative success was defined as extraction of all stone fragments or laser lithotripsy of all stones to less than 2 mm fragments. Moreover, in cases of coexistent renal stones, these stones were also fragmented, simultaneously. After breaking up or removing the stone, a 4.8 F 26 cm internal stent was left in place based on surgeon's discretion. In cases where the ureteral access sheath or flexible ureteroscope could not be advanced up to the proximal ureter, an internal stent was inserted into the ureter; the procedure was called unsuccessful and the intervention was delayed for at least 15 days.

### 2.3. Statistical Analysis

Statistical analysis was performed using the Statistical Package for Social Sciences version 16.0 software (SPSS Inc., Chicago, IL). The measurement data were expressed as mean ± standard error. Student's *t*-test and chi-square test were used for statistical analysis. A value of *p* < 0.05 was considered as statistically significant.

## 3. Results

One hundred and thirty-two patients (86 men and 46 women) were included in this study. Patients' demographics data and stone characteristics are listed in Table 1. Both groups were similar regarding age, gender, BMI, and stone characteristics (Table 1). There were 15 (23%; 15/64) patients with PUS together with renal stones in group 2, all of which were treated with flexible ureteroscope in the same session. Mean renal stone number, renal stone size, and renal stone burden were 1.5 ± 0.2 (1–3), 6.6 ± 0.4 (4–10) mm, and 9.3 ± 1.0 (5–18) mm, respectively, in group 2.

Treatment outcomes are shown in detail in Table 2. A ureteral access sheath was used in 55 (86%; 55/64) patients in group 2. Flexible ureteroscope could have been advanced up into the collecting system without placing access sheath in 5 (8%; 5/64) patients. On the other hand neither flexible ureteroscope nor access sheath could have been advanced up to the proximal ureter in 4 (6%; 4/64) patients. Internal stent placements were left in place in these cases, and the ureteroscopic interventions were successfully completed with flexible ureteroscope 15 days later.

SFR in group 1 was 76.5%. Rigid ureteroscope could have not been advanced up to the proximal ureter in 5 (7%; 5/68) patients. Internal stents were left in place in these cases, and the procedures were completed with flexible ureteroscope after 15 days. Ureteral stones were pushed back to the renal collecting system during the procedure in 8 (12%; 8/68) patients (5 stones during ureteroscopy, 3 stones during lithotripsy), and lithotripsy was completed using flexible ureteroscope. Residual stone fragments larger than 2 mm, most of which were located in the lower calyces on postoperative imaging studies, were detected in 3 (9%; 3/68) patients.

SFR in group 2 was 87.5%, which was not statistically significant compared to that of group 1 (*χ* ^2^ = 0.696; *p* = 0.078). Since neither flexible ureteroscope nor access sheath could have been advanced up to the proximal ureter in 4 (6%; 4/64) patients due to ureteral pathology such as a narrow ureteric lumen and ureteral structures, the procedures were postponed for 15 days. The residual fragments greater than 2 mm remained in 4 (6%; 4/64) patients with PUS and concomitant renal stones in this group, mainly due to intrarenal hemorrhage. There was grade 3 or 4 hydronephrosis in these patients.

Complications were summarized in Table 3. Two major intraoperative complications (ureteral avulsion and ureteral perforation) were seen in group 1 (3%; 2/68). Ureteral avulsion, at the level of ureterovesical junction was seen in a female patient with a PUS 13 mm in size. Ureteral avulsion occurred when reentrance into the ureter was attempted after overdistention of the bladder as a result of prolonged fragmentation. Extravesical ureteroneocystostomy by Lich-Gregoir technique was successfully performed. The patient was discharged uneventfully at postoperative day 4. Another major intraoperative complication was ureteral rupture, seen in a female with a PUS of 10 mm. Ureteral perforation occurred and was repaired by open ureteroureteral anastomosis. The patient was discharged uneventfully at postoperative day 4.

Some minor intraoperative complications including minor ureteral trauma were seen in 6 (9%; 6/68) and 3 (5%; 3/64) patients in groups 1 and 2, respectively. Procedures were not cancelled but internal stents were left in place after the operation in all. Likewise, intraoperative minor hemorrhage was seen in 1 (1%; 1/68) and 4 (6%; 4/64) patients in groups 1 and 2, respectively. None of the patients were given blood transfusions.

Two different postoperative complications (urinary tract infections, and renal colic) were detected (Table 3). Urinary tract infections (Clavien 2) were observed in 2 (3%; 2/68) patients and in 1 (2%; 1/64) patient in groups 1 and 2 and were treated with appropriate antibiotics without hospitalization. Postoperative renal colic after discharge were seen in 4 (6%; 4/68) and 5 (8%; 5/64) patients in groups 1 and 2. Out of these 10 patients, 8 (Clavien 2) were treated with parenteral medications in the emergency setting in both groups (three in group 1; five in group 2). The remaining two patients were treated with internal stent placement (Clavien 3b) due to pain related to hydronephrosis (one in group 1; one in group 2).

## 4. Discussion

The primary goal of complete stone clearance for the management of PUS is to preserve renal function, prevent further stone growth, cure infection, and relieve obstruction [9]. MET is an important treatment option in ureteral stones especially when the stone size is smaller than 5 mm. Since the spontaneous passage rate is only 22%, the majority of PUS need intervention, which depend on various factors including stone size, duration, pain, cost, occurrence of obstruction, and availability of instrument [10, 11]. Currently, the majority of PUS has been treated with ESWL or URS. Nowadays, while ESWL is used in the small (<2 cm) nonimpacted stones, URS is performed in more complicated conditions such as patient with larger and impacted stones [5, 6]. Both procedures have some advantages and disadvantages. ESWL has several advantages including being noninvasive, being safe, not requiring any anesthesia, requiring surgical skills, and being performed as an outpatient setting. On the other hand, URS has a lower retreatment rate and provides immediate stone-free status. But URS requires anesthesia and surgical skills are an invasive procedure and have more complications [3, 5].

Nowadays, ureteroscopic lithotripsy (semirigid or flexible) has been usually performed in the management of PUS. The most important advantage of F-URS compared to SR-URS in treating PUS is to treat coexisting renal stones together with PUS. Multiple stones are detected in 20–25% patients with urolithiasis [12]. In our study, renal stones coexisting with PUS were found in 15 (23%) patients in group 2, and these patients were simultaneously treated with flexible ureteroscope.

There are different success rates of ureteroscopy in the treatment of PUS. Some studies have demonstrated that SFRs of SR-URS have ranged from 51% to 100% in the management of PUS [1, 6, 7, 13–15]. Moufid and colleagues in a retrospective study reported their experience in the management of PUS on 30 patients treated with semirigid ureteroscopic lithotripsy and found that SFRs were 63% [13]. In their study, mean operative time and mean ureteral stone size were 52 ± 17 minutes and 29 ± 1.8 mm, respectively. In our study, rigid ureteroscope was used in 68 patients. In this group (group 1), SFRs, mean operative time, and mean stone size were 76.5%, 34.1 ± 1.5 minutes, and 9.1 ± 0.4 mm, respectively, similar to the literature.

The most important handicap in treating PUS with rigid ureteroscope is pushing the whole stone or stone fragments back into the renal collecting system, which is accepted as a failure and ratio of which between 12% and 25% [4, 16, 17]. In our series, although a stone cone was routinely used in all procedures in group 1, the stones or their fragments were pushed back into the kidney in 8 (12%; 8/68) patients which was due to incomplete blockade of stone cone. We observed that combining F-URS with rigid one resulted in difficulty in the management of PUS because of hemorrhage secondary to high pressure irrigant flow during previous R-URS, which was a contradictory finding in some published series [15, 18]. In our study, hemorrhage, which may blur the vision intraoperatively, was detected in all patients who have been converted to F-URS in group 1. Therefore, when there is a risk of pushing stones or their fragments back, it is better to carry out the procedure with the use of a flexible ureteroscope at the outset, which also prevents bleeding from a previous R-URS.

Flexible ureteroscopic lithotripsy has been performed much more commonly in the treatment of upper urinary system stones lately. In cases where flexible ureteroscope is used in the management of PUS, it has been demonstrated that SFRs are between 79% and 89% [5, 15]. Karadag and colleagues reported their experience in the management of PUS on 61 patients assigned to flexible ureteroscopic lithotripsy and found that SFRs, mean operative time, and mean stone size were 93.4%, 84.1 ± 16.7 minutes, and 11 ± 2.2 mm [7]. In our study, F-URS was performed in 64 patients (group 2). SFRs, mean operative time, and mean stone size were 87.5%, 49.4 ± 2.3 minutes, and 8.9 ± 0.5 mm, respectively.

We have shown that SR-URS was associated with longer hospital stay (*p* = 0.001) and excessive basket catheter use (*p* = 0.001). Longer hospital stays in SR-URS group were due to subsequent open surgery as a result of the major complication. On the other hand, we observed that mean operative time in F-URS group was longer than that of SR-URS group (*p* = 0.001). We think that longer operative times in F-URS group were due to extra time spent for the treatment of coexisting renal stones. However, use of internal stent (*p* = 0.077) and mean internal stenting time (*p* = 0.598) were similar in both groups.

The overall complication rate after URS was reported to be 9–25% [1, 19]. In our series, while intraoperative complication rates were 13% in group 1 and 11% in group 2 (*p* = 0.446; *χ* ^2^ = 0.163), postoperative complication rates in groups 1 and 2 were 9% and 11%, respectively (*p* = 0.453; *χ* ^2^ = 0.166). Ureteral perforation and avulsion are the most serious complications encountered during URS [1, 17, 20]. The incidences of ureteral avulsion and perforation were reported to be 0.1% and 1.7%, respectively in one series [1]. Although complication rates in our study were statistically similar in each group, major complications such as ureteral avulsion and perforation occurred only in group 1. Ureteral avulsion occurred in one patient when reentering into the ureter after overdistention of the bladder as a result of prolonged procedure. This patient was successfully treated with open extravesical ureteroneocystostomy. Ureteral perforation occurred in another patient during stone fragmentation by holmium laser and was treated with open surgery. Both patients in group 1 were discharged from hospital uneventfully at postoperative day 4. Therefore, when rigid ureteroscope is to be used in the treatment of PUS, it should be kept in mind that serious complications may occur.

In times of austerity, cost effectiveness is one of the most important issues in the management of stone disease. The durability and fragility of the rigid ureteroscopes are better than those of the flexible ones. Lifespan of flexible ureteroscopes is limited and the most common cause of scopes' failure is thermal laser damage [21]. 50 consecutive uses from a single flexible ureteroscope were reported by Traxer et al. [21]. In another study by Gurbuz et al., it was reported that the basic cost of flexible ureteroscope per case was $118 [22]. On the other hand, the other ancillary equipments such as fine laser fibers ($24/case), ureteral access sheaths ($231/case), and stone retrieval nitinol baskets ($611/case) that are used F-URS necessitates an extra cost compared to SR-URS [22]. Nonetheless Gurbuz et al. reported that cost for standard F-URS per case was $543 [22]. Due to this reason, when planning to use F-URS for the treatment of PUS, cost issue mentioned above should also be considered.

There are some limitations of our series. Firstly, the present study is limited by both its retrospective nature and being conducted at a single center. Secondly, the small number of patients is another limitation. Randomized prospective and larger series with longer follow-up are necessary to confirm the effectiveness of SR-URS or F-URS. Despite these limitations, to our knowledge, it is the second study to compare the results of SR-URS and F-URS in the management of PUS.

## 5. Conclusions

Our data showed that F-URS is safer and less invasive than SR-URS in patients with PUS. Despite the fact that there is no statistically significant difference in the efficacy of either technique, there was a trend towards the better performance of F-URS. Therefore, we recommend F-URS in the management of PUS as a first-line treatment option in select cases of proximal ureteral calculi.

## Figures and Tables

**Table 1:** Patients' demographics data and stone characteristics.

	Group 1 (R-URS)	Group 2 (F-URS)	*p* value
Gender (M/F)	41/27	45/19	0.554
Mean patient age (year)	38.2 ± 1.3	39.9 ± 1.3	0.991
(Range)	(21–74)	(21–75)	
Mean BMI (kg/mm^2^)	27.1 ± 0.4	27.8 ± 0.5	0.136
(Range)	(21–41)	(22–45)	
Mean ureteral stone number (*n*)	1.1 ± 0.1	1.0 ± 0.1	0.353
(Range)	(1-2)	(1–5)	
Mean ureteral stone size (mm)	9.1 ± 0.4	8.9 ± 0.5	0.599
(Range)	(5–20)	(5–20)	
Mean ureteral stone burden (mm)	9.8 ± 0.4	9.2 ± 0.4	0.607
(Range)	(5–22)	(6–23)	
Laterality (R/L)	30/38	28/36	0.553

**Table 2:** Operative and postoperative data.

	Group 1 (R-URS)	Group 2 (F-URS)	*p* value
Mean operative time (min)	34.1 ± 1.5	49.4 ± 2.3	**0.001**
(Range)	(10–75)	(20–90)	
Mean hospital stay (hour)	28.0 ± 1.9	24.5 ± 1.1	**0.001**
(Range)	(12–96)	(12–72)	
Use of basket catheter (*n*)	52/68	30/64	**0.001**
Use of internal stent (*n*)	32/68	39/64	0.077
Mean internal stenting time (day)	23.2 ± 2.6	27.3 ± 2.3	0.598
(Range)	(7–90)	(3–90)	
SFR	76.5%	87.5%	0.078

**Table 3:** Intraoperative and postoperative complications.

	Group 1 (R-URS)	Group 2 (F-URS)	*p* value
Intraoperative	9 (13%)	7 (11%)	0.446
(i) Ureteral avulsion	1	—	
(ii) Ureteral perforation	1	—	
(iii) Minor ureteral trauma	6	3	
(iv) Minor hemorrhage	1	4	
Postoperative	6 (9%)	7 (11%)	0.453
(i) Urinary tract infection	2	1	
(ii) Renal colic	4	6	
